# Plasmid transfection influences the readout of nonsense-mediated mRNA decay reporter assays in human cells

**DOI:** 10.1038/s41598-017-10847-4

**Published:** 2017-09-06

**Authors:** Jennifer V. Gerbracht, Volker Boehm, Niels H. Gehring

**Affiliations:** Institute for Genetics, Department of Biology, University of Cologne, 50674 Cologne, Germany

## Abstract

Messenger RNA (mRNA) turnover is a crucial and highly regulated step of gene expression in mammalian cells. This includes mRNA surveillance pathways such as nonsense-mediated mRNA decay (NMD), which assesses the fidelity of transcripts and eliminates mRNAs containing a premature translation termination codon (PTC). When studying mRNA degradation pathways, reporter mRNAs are commonly expressed in cultivated cells. Traditionally, the molecular mechanism of NMD has been characterized using pairs of reporter constructs that express the same mRNA with (“PTC-containing mRNA”) or without (“wild-type mRNA”) a PTC. Cell lines stably expressing an NMD reporter have been reported to yield very robust and highly reproducible results, but establishing the cell lines can be very time-consuming. Therefore, transient transfection of such reporter constructs is frequently used and allows analysis of many samples within a short period of time. However, the behavior of transiently and stably transfected NMD constructs has not been systematically compared so far. Here, we report that not all commonly used human cell lines degrade NMD targets following transient transfection. Furthermore, the degradation efficiency of NMD substrates can depend on the manner of transfection within the same cell line. This has substantial implications for the interpretation of NMD assays based on transient transfections.

## Introduction

The transfer of correct genetic information from the DNA to the translated protein is essential for the survival of a cell. Therefore, cellular quality control mechanisms exist, which detect and degrade aberrant transcripts^1^. Nonsense-mediated mRNA decay (NMD) is a translation-coupled quality control pathway in eukaryotic cells that targets mRNAs carrying a premature termination codon (PTC)^2^. Aberrant translation termination at the PTC causes stalling of the ribosome and the assembly of a surveillance complex, which recruits RNA degradation enzymes^3^. This prevents the production of truncated proteins with a potentially dominant-negative effect^4^. NMD is an important modulator of the phenotype of genetic disorders, as nonsense mutations represent 20% of disease-associated single base pair substitutions affecting gene coding regions^5^. Moreover, NMD acts as a general modulator of gene expression, since many transcripts that have arisen from alternative splicing or contain an upstream open reading frame or long 3′ UTR are endogenous NMD targets^6^.

Numerous proteins and factors involved in NMD have been identified, but many questions regarding the exact mechanism by which NMD degrades mRNAs remain to be answered^7^. In the study of NMD, reporter mRNAs with and without a PTC are utilized to monitor the degradation of transcripts in standard human cell lines such as HeLa and HEK-293 cells. When introduced into the cell via transient transfection, these transcripts are expressed from a high number of extrachromosomal plasmids. Alternatively, isogenic cell lines stably expressing the reporter constructs can be established. When using a system such as Flp-In T-REx, the target gene is inserted at a single site in the cell line of choice. While overall expression levels of the reporter mRNAs are lower, previous studies suggest that more pronounced and robust results can be achieved using this approach^8^. However, generation of stable cell lines can be laborious and especially when a high number of reporter constructs are systematically compared, transient transfections yield faster results.

While studying NMD using many different cell lines, reporter constructs and transfection methods, we observed notable discrepancies. Since similar experimental designs are widely used in the field, we sought to systematically analyze the NMD efficiency in standard human cell lines. We found that not all commonly cultured human cells effectively degrade NMD targets when reporter mRNAs are expressed from extrachromosomal plasmid DNA. Additionally, for the widely used HEK-293 cells we observed differential NMD efficiency depending on whether stable or transient transfections have been performed.

## Results and Discussion

### Differential NMD efficiency in transiently transfected HeLa and HEK-293 cells

**Figure 1:**
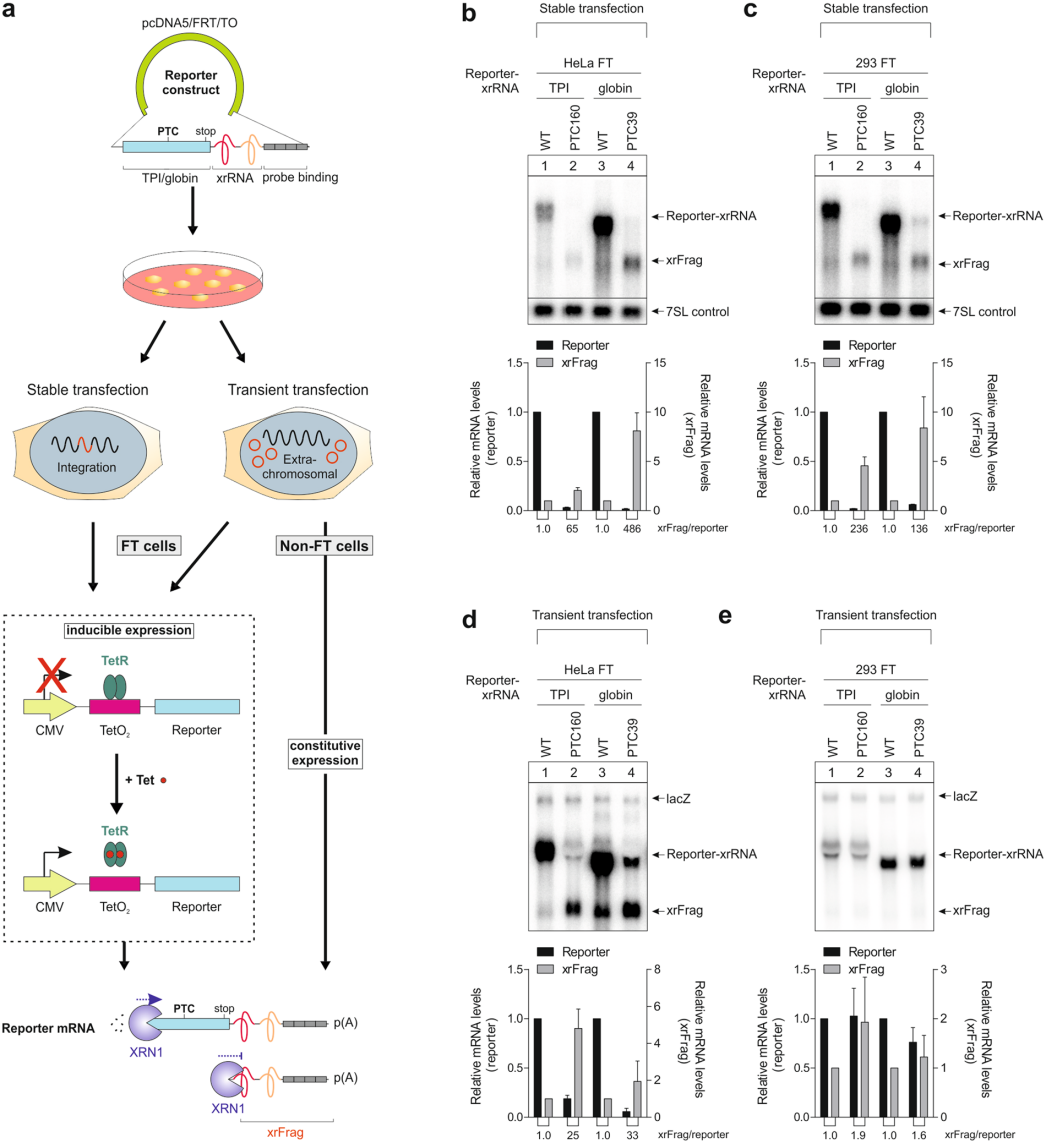
Transiently transfected 293 FT cells show a strongly reduced NMD efficiency in comparison to stably transfected cells. (**a**) Schematic representation of the experimental setup. The reporter constructs are generated by cloning the gene of interest into the pcDNA5/FRT/TO vector. The blue bar represents the coding sequence of triosephosphate isomerase (TPI) or β-globin with or without a premature termination codon (PTC) and with a normal stop codon (stop). Downstream, a viral XRN1-resistant sequence (xrRNA) is present that allows the detection of decay intermediates resulting from 5′–3′ exonucleolytic decay. The grey boxes correspond to four repeats of a northern blot probe binding site. Cells are transfected with these constructs using either a stable or transient transfection system. Flp-In T-REx (FT) cells constitutively express a Tet repressor (TetR) that blocks transcription of the reporter by binding to Tet operators (TetO_2_) present downstream of the cytomegalovirus (CMV) promoter in the reporter construct. Therefore, FT cells are induced with tetracycline(Tet)/doxycycline. In non-FT cells, the reporter is always expressed. Unstable reporter mRNAs are degraded with participation of the cellular 5′–3′ exonuclease XRN1. Full-length reporter levels and decay fragment (xrFrag) caused by stalling of XRN1 at the xrRNA are detected via northern blotting. p(A): poly(A) tail. (**b**,**c**) Expression of reporter mRNAs in HeLa FT (**b**) and 293 FT (**c**) stable cell lines was induced with doxycycline for 24 h and the reporter levels detected by northern blotting. The lower band (xrFrag) corresponds to a 5′–3′ exonucleolytic decay intermediate. Endogenous 7SL RNA levels were detected and quantified on the same blot and are shown as control. The mean values ± SD (n = 3) for relative transcript levels were quantified and the PTC values normalized to the wild-type control. The xrRNA/reporter ratio is indicated below the graph. (**d**,**e**) HeLa FT (**d**) and 293 FT (**e**) cells were transiently transfected with 3 µg plasmid DNA per 6-well and transcription was induced with doxycycline for 24 h. Reporter mRNA levels were analyzed by northern blotting. LacZ was co-expressed and serves as a transfection control. The mean values ± SD (n = 3) were quantified and the PTC values normalized to the wild-type control. The xrRNA/reporter ratio is shown below the graph.

In order to determine the degradation efficiency of transfected (i.e. exogenous) NMD targets, we have developed an experimental system to compare different cell lines and transfection systems (Fig. 1a). First, we established inducible HeLa and HEK-293 Flp-In T-REx stable cell lines (referred to as HeLa FT and 293 FT, respectively) expressing triosephosphate isomerase (TPI) or globin reporter mRNA with or without a PTC. In both types of cells, the levels of PTC-containing full-length reporter mRNA were markedly reduced (Fig. 1b,c), indicating that these transcripts are efficiently degraded by NMD. An additional faster migrating band detected by northern blotting corresponds to a decay fragment (xrFrag) containing an xrRNA sequence, which is resistant to the 5′–3′ exonuclease XRN1^8^. The accumulation of xrFrags correlates with mRNA decay activity and serves as a readout for mRNA degradation pathways involving 5′–3′ decay^8^. When we transiently transfected the TPI and globin NMD reporter constructs in HeLa FT cells, the PTC-containing reporter mRNA was also strongly reduced (Fig. 1d). Strikingly, we did not observe differences between the WT and PTC constructs in transiently transfected 293 FT cells: the expression levels of both mRNAs were comparable and no xrFrags accumulated (Fig. 1e). This result was surprising since the reporter mRNA was expressed from the same pcDNA5/FRT/TO plasmid under control of the CMV promoter that was used for generating the stable cell lines. It has been reported before that NMD efficiency can vary between different strains of cells, for example for the HeLa cell model system^9^ or epithelial cells of cystic fibrosis patients^10^. NMD efficiency has also been described to vary between different murine tissues^11^. In contrast, the observation made here is that the degradation of an NMD-target depends on the manner of transfection and not on the inherent characteristic of the cell.

### NMD deficiency of transiently transfected HEK-293 cells is independent of transfection reagent and plasmid amount

**Figure 2:**
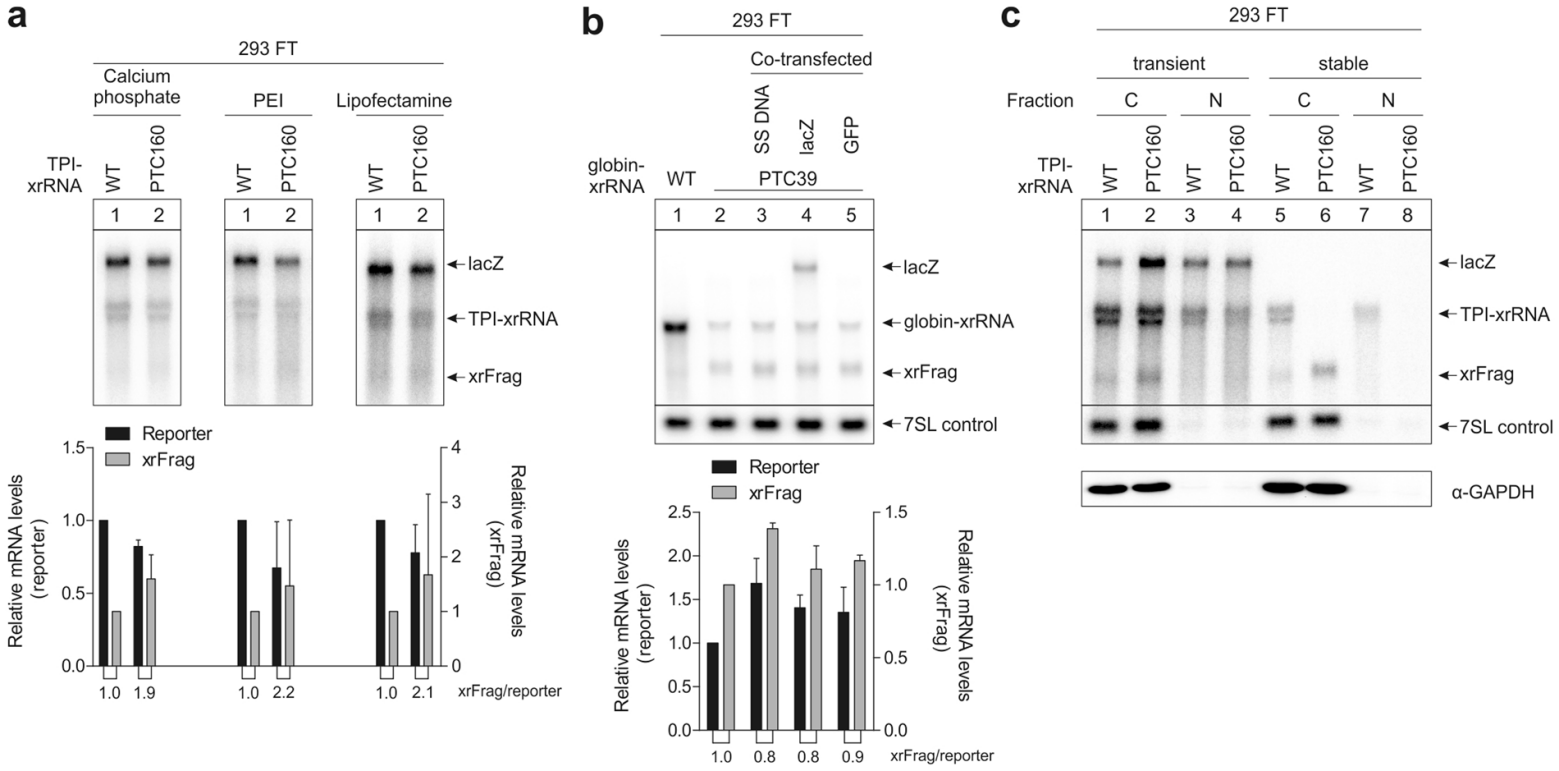
The reduced NMD efficiency in transiently transfected 293 FT cells is not due to transfection conditions. (**a**) Northern blots of the indicated reporter mRNAs. 293 FT cells were transiently transfected with 0.3 µg of plasmid DNA per 6-well using different transfection reagents. LacZ was co-transfected as a control mRNA. The mean reporter values ± SD (n = 3) were quantified and the PTC-reporter levels normalized to the wild-type control. The xrRNA/reporter ratio is indicated below the graph. (**b**) RNA samples were extracted from stably transfected 293 FT cells and analyzed by northern blotting. The samples shown in Lanes 3–5 have additionally been transiently transfected with salmon sperm DNA (SS DNA), lacZ and a GFP expression plasmid, respectively. Endogenous 7SL RNA levels were detected and quantified on the same blot and are shown as control. The mean values ± SD (n = 3) were quantified and the values of the co-transfected samples normalized to the untransfected PTC control. The xrRNA/reporter ratio is indicated below the graph. (**c**) Northern blot of RNA extracted from the indicated fractions of 293 FT cells transfected with 0.3 µg of plasmid DNA per 6-well. Co-transfected lacZ is shown as a control. Endogenous 7SL RNA levels were detected on the same blot as control. C: cytoplasmic fraction, N: nuclear fraction. Successful fractionation was confirmed by the cytoplasmic marker GAPDH and the localization of the 7SL control RNA.

Our initial observation was made using the calcium phosphate precipitation method for transient transfections. Although calcium phosphate-based transfections are commonly used and led to robust NMD readout in HeLa cells, we wanted to test whether the transfection method had an influence on the behavior of the reporter mRNA in the cell. To this end, the experiment was repeated using other commonly used reagents to transiently transfect cultured cells. However, no substantial differences between wild-type and PTC mRNAs in 293 FT cells were observed when the constructs were transfected using polyethylenimine (PEI) or Lipofectamine 2000 (Fig. 2a). Different cellular stress pathways have been previously implicated in the regulation of NMD efficiency^12^. To rule out that the observed inability to degrade an NMD reporter was due to cellular stress caused in response to transient transfections, a mock transfection of 293 FT cells stably expressing globin PTC39 mRNA was performed (Fig. 2b). When the stable cell lines were transiently co-transfected with salmon sperm DNA, the mRNA expressed from the stably integrated PTC39 construct was still efficiently degraded (Fig. 2b, lane 3). The same observation was made when the cells were co-transfected with the lacZ expression plasmid (Fig. 2b, lane 4) as well as with a GFP-expressing plasmid (Fig. 2b, lane 5, GFP expression was confirmed by microscopy), which were both included in our transient transfections as controls.

For functional NMD, the transcript must be exported to the cytoplasm and be actively translated. We confirmed that the reporter mRNA is exported to the cytoplasm via subcellular fractionation (Fig. 2c). Furthermore, the translatability of these transcripts was assessed by expressing GFP from transiently transfected plasmids in 293 FT cells and confirming GFP levels by microscopy.

**Figure 3:**
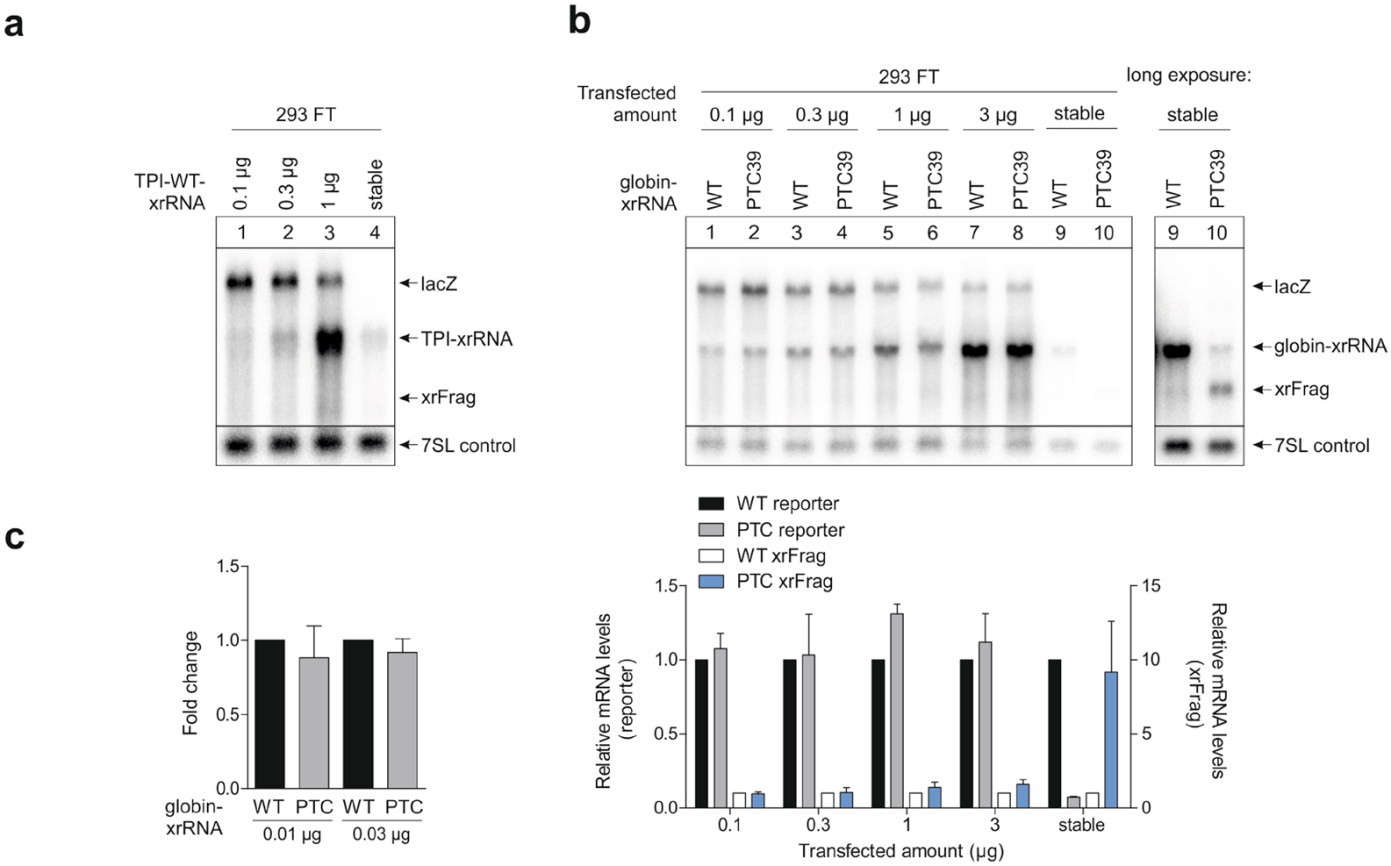
NMD efficiency in transiently transfected 293 FT cells does not depend on the amount of transfected plasmid DNA. (**a**) Lanes 1–3: 293 FT cells were transfected with the indicated amount of plasmid DNA per 6-well and the mRNA was analyzed by northern blotting. LacZ was co-transfected as a control. Lane 4: 293 FT cells stably transfected with the indicated construct were induced for 24 h. Endogenous 7SL RNA levels were detected on the same blot and are shown as control. (**b**) Lanes 1–8: 293 FT cells were transfected with the indicated amount of plasmid DNA per 6-well and the mRNA was analyzed by northern blotting. LacZ was co-transfected as a control. Lanes 9–10: 293 FT cells stably transfected with the indicated construct were induced for 24 h. Endogenous 7SL RNA levels were detected and quantified on the same blot and are shown as control. The mean values ± SD (n = 3) of the signals were quantified and the PTC-values normalized to the corresponding wild-type control. (**c**) 293 FT cells were transiently transfected with the indicated amount of plasmid DNA per 6-well and expression levels were measured by qPCR. The reporter mRNA levels were normalized to the co-transfected GFP control. The bars represent mean fold changes of PTC-containing reporter vs. wild-type reporter levels ± SD (n = 3).

In stably transfected FT cells, the gene of interest is integrated at a single site in the genome in every cell and the total cell population is isogenic. In transiently transfected cells however, the amount of plasmid DNA and expressed mRNA varies between cells. To exclude that the apparent inhibition of NMD results from overloading individual cells using the transient system, we transfected 293 FT cells with lower amounts of plasmid DNA. We estimated that 0.1–0.3 µg of transiently transfected plasmid DNA yields expression levels that are comparable to the amount expressed in stable cell lines after 24 h of induction (Fig. 3a). When transfecting 293 FT cells using these reduced concentrations, still no degradation of the PTC-containing mRNA could be detected down to 0.1 µg of transfected plasmid DNA (Fig. 3b). Although the average mRNA levels in stable cell lines and cells transfected with 0.1 µg plasmid DNA were comparable, individual transiently transfected cells might express more than the average stably transfected cell. Therefore, we transfected lower amounts of DNA (0.01 and 0.03 µg) and quantified reporter mRNA expression by qPCR, since expression levels were too low to be detectable by northern blotting (Fig. 3c). Even when using these minimal amounts of transfected reporter DNA, no difference could be detected between wild-type and PTC-containing reporter mRNA.

Collectively, these results show that the inability of transiently transfected 293 FT cells to degrade NMD reporter mRNAs is independent of transfection reagent and the amount of transfected plasmid DNA. This is in line with the fact that HeLa cells, which have been transiently transfected with a high amount of plasmid DNA, degrade PTC-containing reporters very efficiently (Fig. 1d).

### Not all degradation pathways are impaired in transiently transfected HEK-293 cells

**Figure 4:**
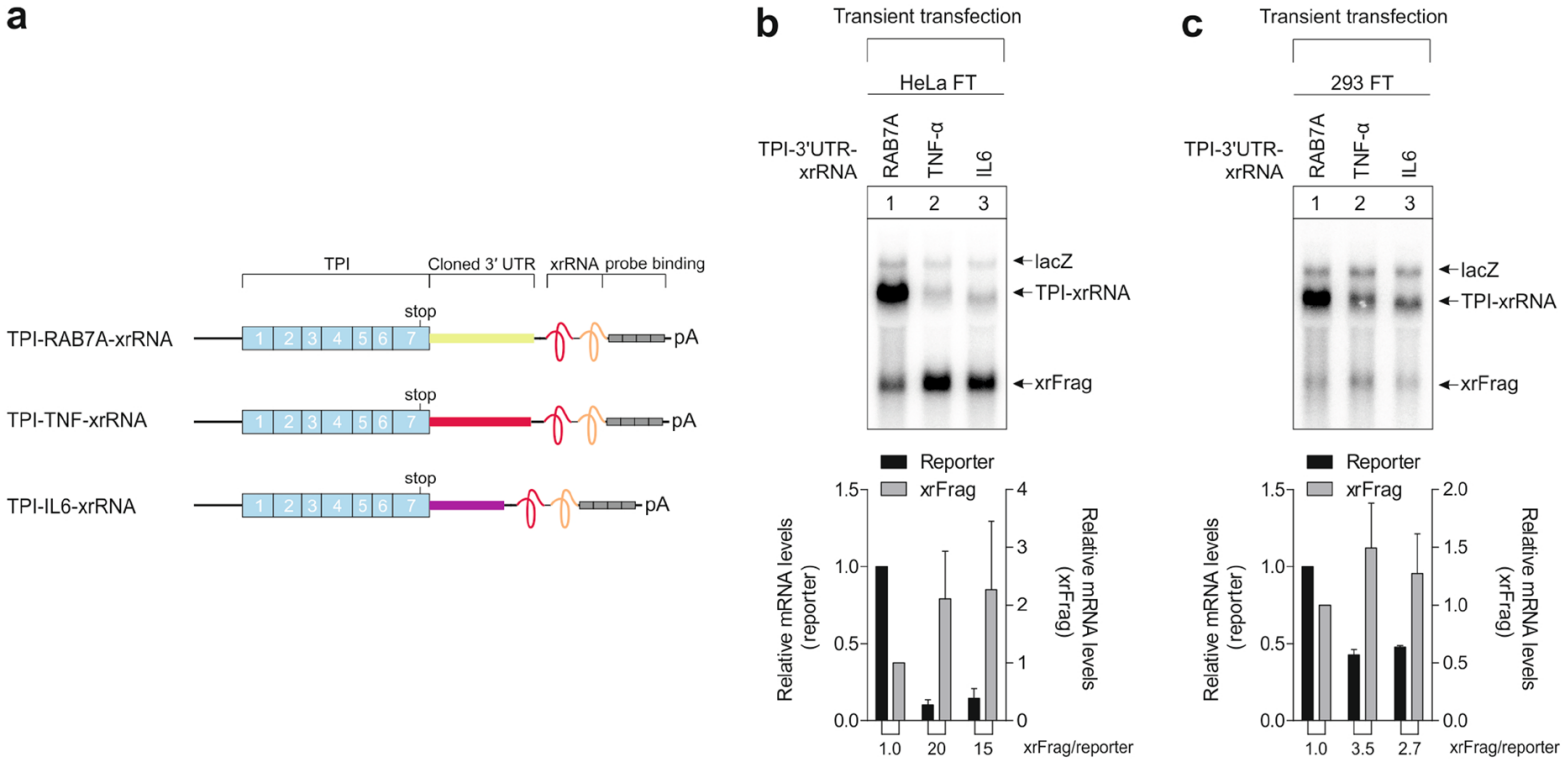
Degradation of unstable reporter mRNAs in 293 FT cells. (**a**) Schematic representation of the reporter constructs as in Fig. 1a. Downstream of the TPI gene, the 3′ UTRs of RAB7A (control), TNF-α and IL6 were cloned. (**b**,**c**) HeLa FT (**b**) and 293 FT (**c**) cells were transfected with 3 µg plasmid DNA of the indicated reporter per 6-well. The reporter levels were analyzed by northern blotting. LacZ was co-transfected as a transfection control. The mean reporter values ± SD (n = 3) were quantified and the cytokine reporter mRNAs normalized to the RAB7A control. The xrFrag/reporter ratio was calculated and is indicated below the graph.

To investigate whether 293 FT cells exhibit a general defect to efficiently degrade unstable mRNAs expressed from transiently transfected plasmids, we studied reporter mRNAs containing the 3′ UTRs from the cytokines TNF-α and Interleukin-6 (IL6) in HeLa and 293 FT cells (Fig. 4a). These 3′ UTRs contain several degradation-promoting sequences such as AU-rich elements and a decay-inducing stem-loop^13^. Interestingly, both reporter mRNAs were degraded in 293 FT cells, although the reduction of full length mRNA as well as accumulation of xrFrags was less pronounced than in HeLa FT cells (Fig. 4b,c).

### Variable degradation of transiently transfected NMD reporters in other human cell lines

**Figure 5:**
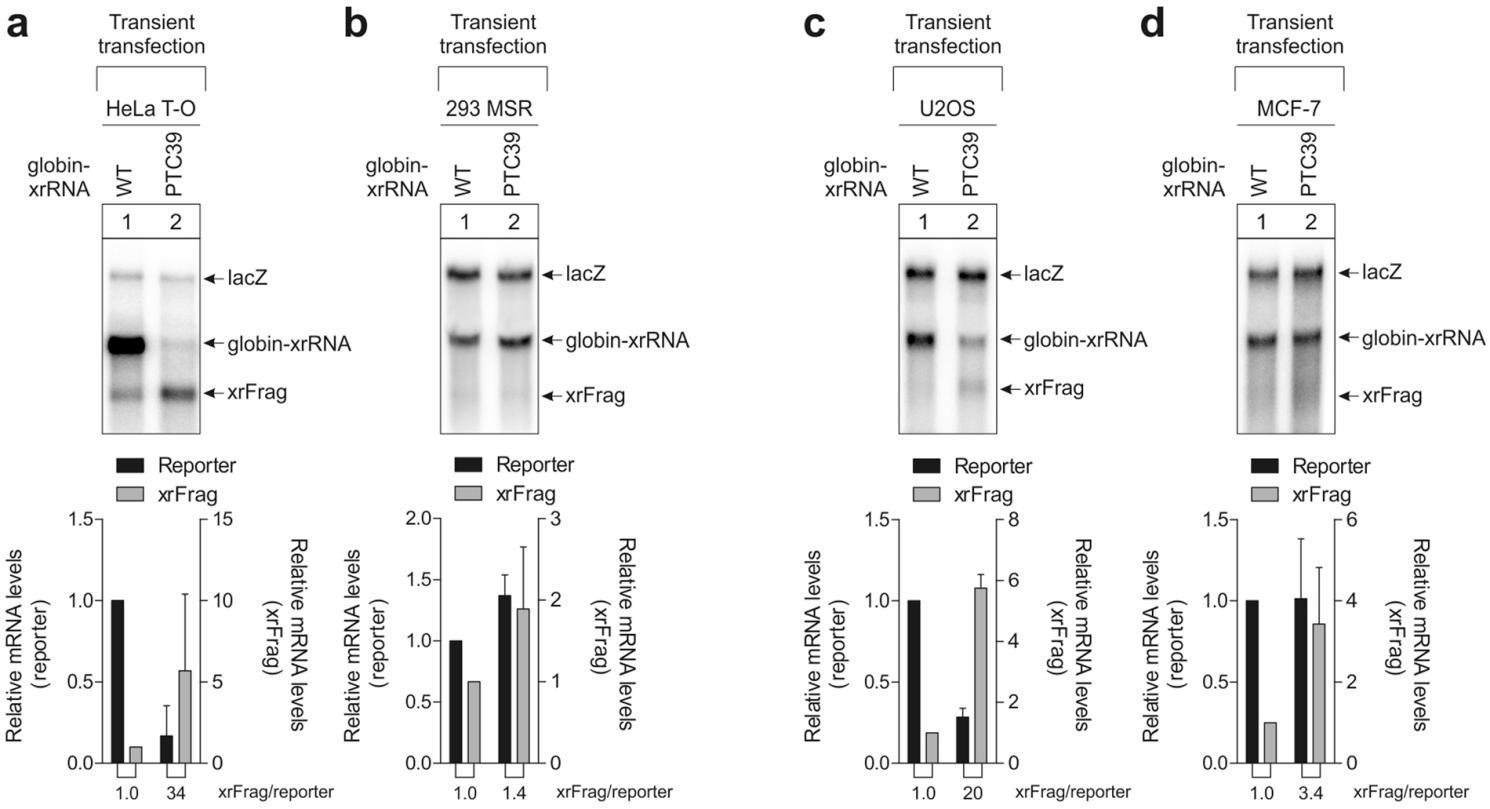
Differential NMD efficiency in transiently transfected human cells. (**a**–**d**) HeLa Tet-Off (HeLa T-O, (**a**), GripTite 293 MSR (**b**), U2OS (**c**) and MCF-7 (**d**) cells were transfected with 0.3 µg plasmid-DNA per 6-well and the RNA was extracted and analyzed by northern blotting. HeLa T-O, 293 MSR and U2OS cells were transfected using calcium phosphate transfection and MCF-7 using jetPRIME transfection reagent. LacZ was co-transfected as a control. The mean values of reporter ± SD (n = 3) were quantified and the PTC-reporter levels normalized to the wild-type control. The xrRNA/reporter ratio is indicated below the graph.

In order to establish whether the phenomenon of impaired NMD efficiency of transiently transfected cells is limited to 293 FT cells, more human cell lines were analyzed (Fig. 5). First, other strains of HeLa and HEK-293 cells were examined, which recapitulated the results obtained from their Flp-In T-REx counterparts (Fig. 5a,b). Next, further commonly used cancer cell lines were investigated. In U2OS cells that have been transiently transfected with globin reporter mRNA with or without a PTC, the full-length reporter levels were reduced, whereas the xrFrag accumulated, suggesting efficient NMD (Fig. 5c). In contrast, in MCF-7 cells transfected with the same construct, the full-length reporter mRNA levels were not reduced (Fig. 5d), analogous to the observation made in HEK-293 cells. These findings demonstrate that reduced NMD of transiently expressed reporter mRNAs is not limited to HEK-293 cells and that caution should be taken when investigating NMD in human cell lines using a transient transfection system.

## Conclusion

To the best of our knowledge, this is the first comparison of stably vs. transiently transfected NMD reporters in cultured human cells. Of the six different cells lines tested in this study, three (293 FT, 293 MSR and MCF-7) did not show efficient degradation of NMD-reporter mRNAs in transient transfection experiments. Importantly, HEK-293 and MCF-7 cells are widely used and easy to handle human cell lines and may therefore be chosen for transient transfections by a substantial number of laboratories. Considering that we have analyzed only a small number of cell lines until now, it may be premature to conclude that NMD substrates are generally less efficiently degraded when expressed from transiently transfected reporter constructs. Nonetheless, our results raise the concern that the robustness of NMD assays using transfected reporters may be influenced by a previously unknown interference of transient transfections with the NMD competence of certain cell lines. Currently, the reason for the impaired NMD of transiently transfected reporters in HEK-293 cells and MCF-7 cells is unclear. We have confirmed that mRNAs expressed from extrachromosomal plasmids are transported to the cytoplasm and efficiently translated, two known requirements for the activation of NMD. It is conceivable that transiently expressed mRNAs are unable to recruit NMD-promoting messenger ribonucleoprotein (mRNP) components. However, the identity of such mRNP components remain to be determined in future experiments. Furthermore, we cannot exclude that transiently expressed mRNAs are in general more resistant to mRNA degradation in HEK-293 cells. Although the decay of TNF-α and Interleukin-6 3′ UTR-containing reporter mRNAs is more pronounced than that our NMD reporter mRNAs, it not does not match the turnover efficiency of reporter mRNAs expressed from stably integrated constructs. Taken together, mRNAs/mRNPs expressed by transient transfections may exhibit a differential molecular composition or contain certain modifications that render them more resistant to mRNA turnover than mRNAs transcribed from chromosomal insertions. Therefore, we strongly suggest to assess NMD efficiencies using independent methods. Such initial tests will be particularly important when previously uncharacterized cell lines are used or when NMD efficiencies of different cells are compared.

## Materials and Methods

### Plasmid constructs and cell culture

The plasmid constructs β-globin-WT and -PTC39; TPI-WT and -PTC160; TPI-RAB7A, -TNF and -IL6 containing an XRN1-resistant structure from the Murray Valley encephalitis virus were generated by cloning the respective DNA fragments^8^ into the pcDNA5/FRT/TO vector (Thermo Fisher Scientific). The mVenus and lacZ control plasmid were constructed by inserting the respective DNA fragments into the pCI-neo vector (Promega) as described previously^14^.

HEK-293 Flp-In T-REx (293 FT; Thermo Fisher Scientific), HeLa Flp-In T-REx (HeLa FT; established by Elena Dobrikova and Matthias Gromeier, Duke University Medical Center), HeLa Tet-Off (Clontech), GripTite 293 MSR (Thermo Fisher Scientific), U2OS and MCF-7 cells were cultured in Dulbecco’s Modified Eagle’s Medium (Gibco, Life Technologies) supplemented with Penicillin-Streptomycin and 9% FBS. The cells were maintained at 37 °C, 5% CO_2_ and 90% humidity.

### Establishment of stable cell lines

For stable transfections, HeLa FT or 293 FT cells were seeded at a density of 2.8 × 10^5^ cells/well in 6-well plates 24 h before transfection. In each well, 2.5 µg of the pcDNA5/FRT/TO vector with the gene of interest and 0.5 µg of the pOG44 vector expressing the Flp recombinase were transfected with the calcium phosphate method. 48 h after transfection, the cells were transferred to 10 cm culture dishes and selected with a hygromycin concentration of 100 µg/ml (293 FT cells) or 150 µg/ml (HeLa FT cells). Expression of stable cell lines was induced with 1 µg/ml doxycycline for 24 h.

### Transient plasmid transfections

2.8 × 10^5^ cells were seeded in 6-well plates 24 h before transfection. If not indicated otherwise, the cells were transfected using the calcium phosphate method. The indicated amount of reporter plasmid was co-transfected with 0.5 µg of the mVenus expression plasmid and 3 µg of the lacZ control plasmid. Polyethylenimine (PEI) transfections were performed according to the jetPEI protocol (Polyplus transfection). Transfections using jetPRIME (Polyplus transfection) and Lipofectamine 2000 (Thermo Fisher Scientific) were performed according to the manufacturer’s instructions. Due to the presence of Tet repressor binding sites in the pcDNA5/FRT/TO vector and the constitutive expression of the Tet repressor in the FT cell lines, expression from transiently transfected pcDNA5/FRT/TO reporter plasmids in these cells was induced by supplementing the medium with 1 µg/ml doxycycline for 24 h.

### Subcellular fractionation

The cells were harvested in polysome buffer (10 mM NaCl, 10 mM MgCl_2_, 10 mM Tris-HCl pH 7.4, 1% Triton X-100, 1% Na-deoxycholate and 1 mM DTT). The cytoplasmic and nuclear fractions were separated by centrifugation.

### RNA extraction and northern blotting

The cells were harvested in peqGOLD TriFast reagent (VWR) and total RNA extraction was performed as recommended by the manufacturer’s protocol. 2.5 µg of total RNA were resolved on a 1% agarose/0.4 M formaldehyde gel using the tricine/triethanolamine buffer system as described^15^ followed by transfer on a nylon membrane (Roth) in 10x SSC. The blots were incubated overnight at 65 °C in Church buffer containing [α-^32^P]-GTP body-labeled RNA probes for detection of the reporter and lacZ control RNA. Endogenous 7SL RNA was detected by a 5′-^32^P-labeled oligonucleotide (5′-TGCTCCGTTTCCGACCTGGGCCGGTTCACCCCTCCTT-3′). The blots were visualized and quantified using the Typhoon FLA 7000 (GE Healthcare) and ImageQuant TL 1D software.

### Western blot analysis

Proteins were extracted using peqGOLD TriFast reagent (VWR), separated by SDS-PAGE gel electrophoresis and transferred to nitrocellulose membranes. The following antibodies were used: anti-GAPDH (Santa Cruz Biotechnology), peroxidase-coupled secondary anti-mouse (Jackson ImmunoResearch). Detection was performed with Western Lightning Plus-ECL (PerkinElmer) and myECL Imager (Thermo Fisher Scientific).

### Quantitative real-time PCR analysis

1 µg of total RNA was reverse-transcribed in a 20 µl reaction volume with an oligo dT primer (5′-TTTTTTTTTTTTTTTTTTTTVNN-3′, 10 µM final concentration) using the ProtoScript II Reverse Transcriptase (NEB) according to the manufacturer’s instructions. PCRs were performed using one-tenth of the reverse transcription reaction with GoTaq qPCR Master Mix (Promega) and the CFX96 Touch Real-Time PCR Detection System (Bio-Rad). The reactions were performed in triplicates and the average C_t_ (Threshold cycle) value was calculated. Globin mRNAs levels were normalized to mVenus mRNA levels. For each primer, a 2-fold standard dilution was performed to calculate the primer efficiencies, which ranged between 90–100%. The fold changes were calculated using the ΔΔC_t_ method^16^. The mean fold changes were calculated from three biologically independent experiments. Primer sequences were: 5′-CAGGCTGCTGGTGGTCTAC-3′ and 5′-CGTGCAGCTTGTCACAGTG-3′ (globin mRNA); 5′-CCATCTTCTTCAAGGACGAC-3′ and 5′-TGATATAGACGTTGTGGCTG-3′ (mVenus mRNA).

### Data availability statement

The authors declare that the data supporting the findings of this study are available within the paper.

## Electronic supplementary material

Supplementary information
