# Supplementary material for: Plasmid transfection influences the readout of nonsense-mediated mRNA decay reporter assays in human cells
Source: Sci Rep. 2017 Sep 6;7:10616. doi: 10.1038/s41598-017-10847-4 (PMC5587671; doi:10.1038/s41598-017-10847-4)
Supplement: Supplementary file 1 — Supplementary information [file 41598_2017_10847_MOESM1_ESM.pdf]

Supplementary Information

Plasmid transfection influences the readout of nonsense-mediated mRNA decay reporter assays in human cells

Jennifer V. Gerbracht, Volker Boehm and Niels H. Gehring

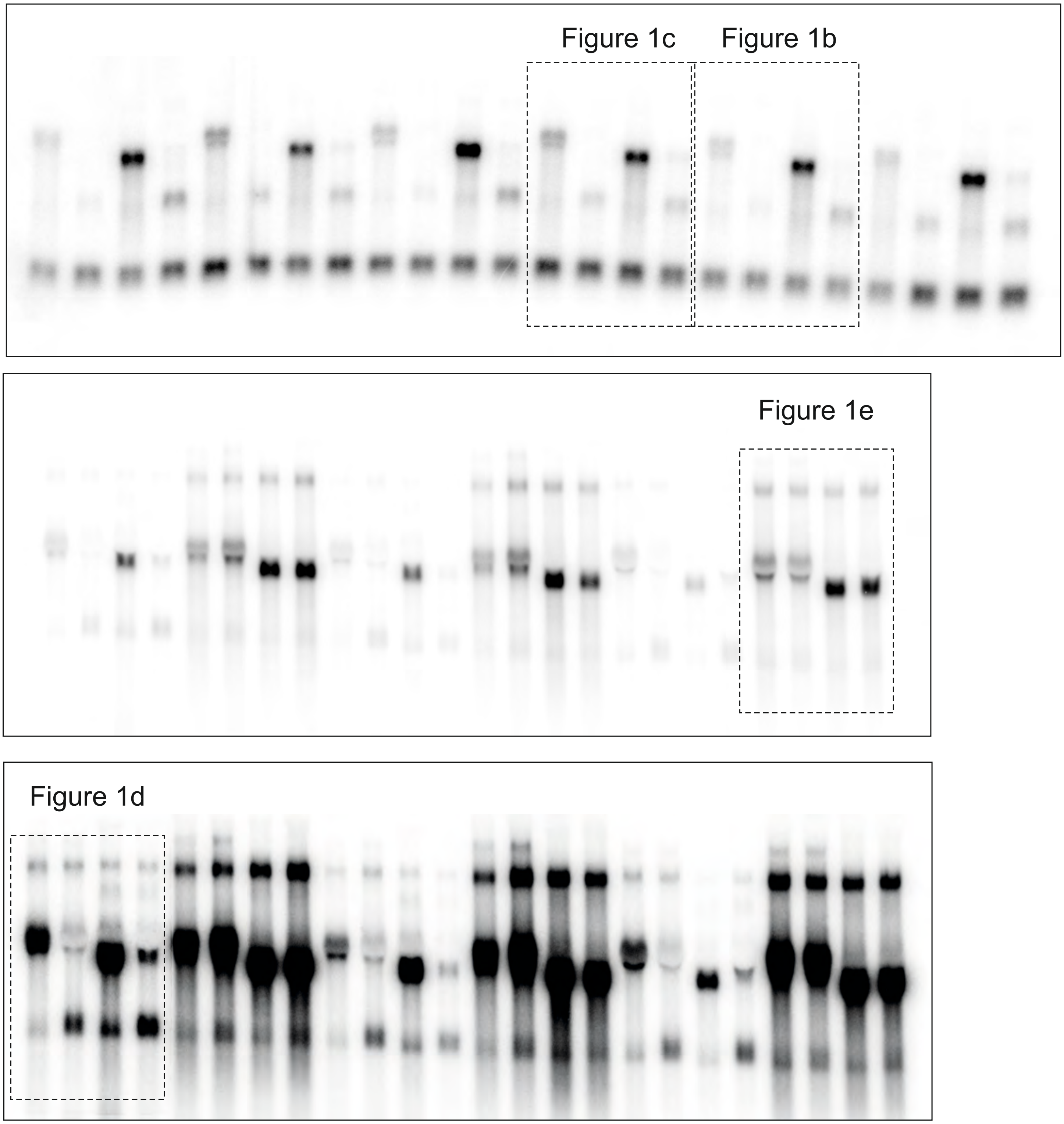

Supplementary Figure 1

Uncropped blots of Figures 1-5 as indicated.

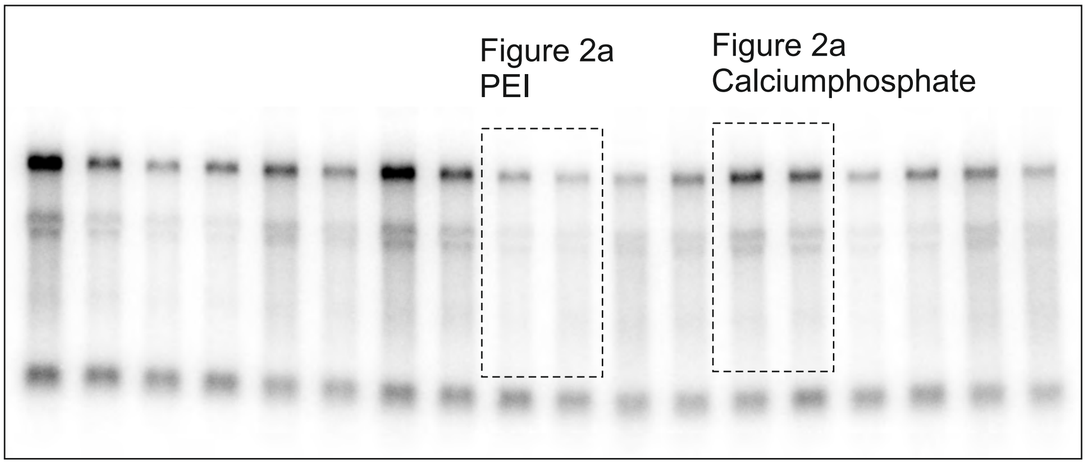

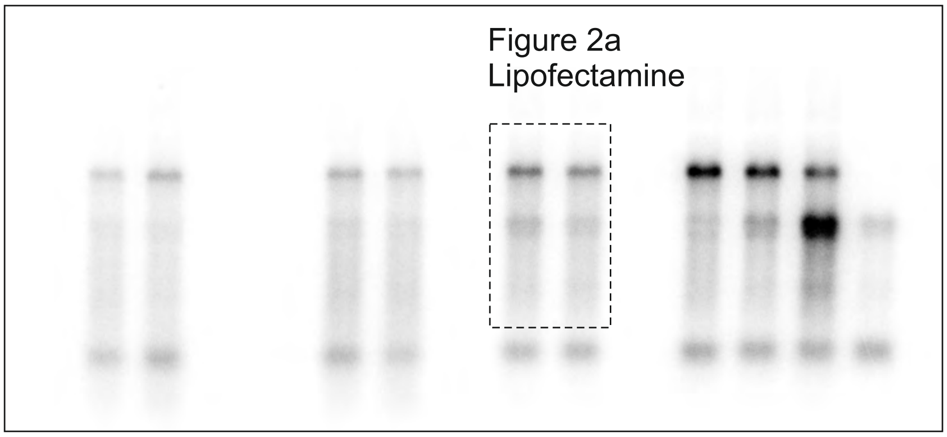

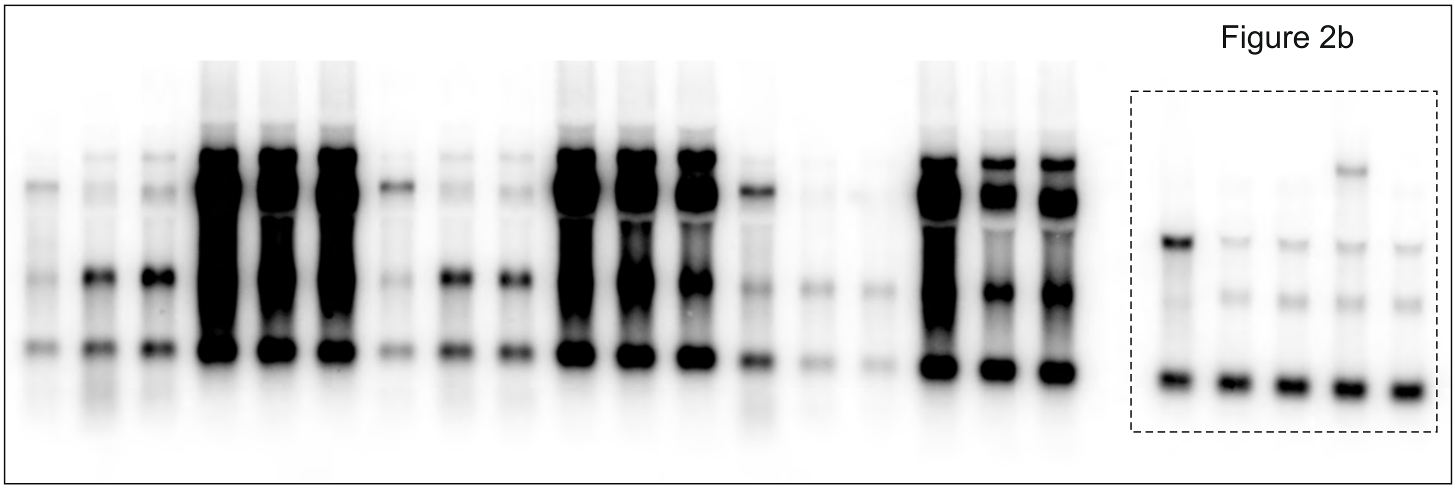

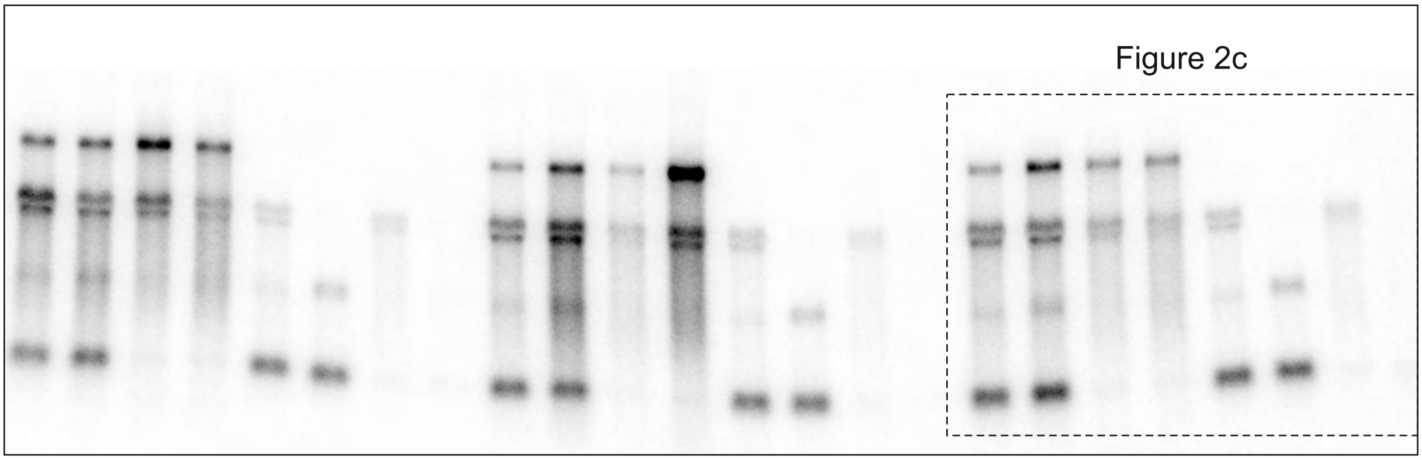

Figure 2c

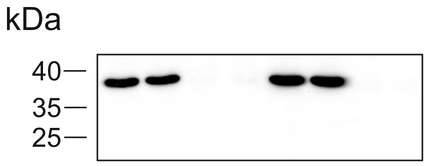

Supplementary Figure 1, continued

Uncropped blots of Figures 1-5 as indicated.

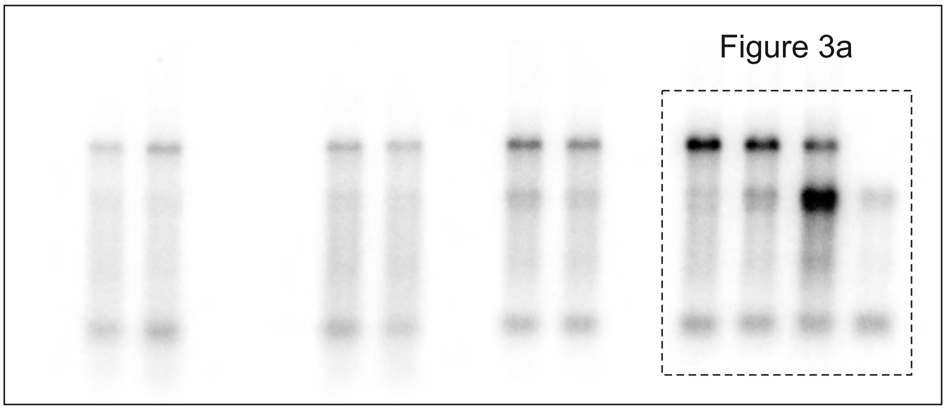

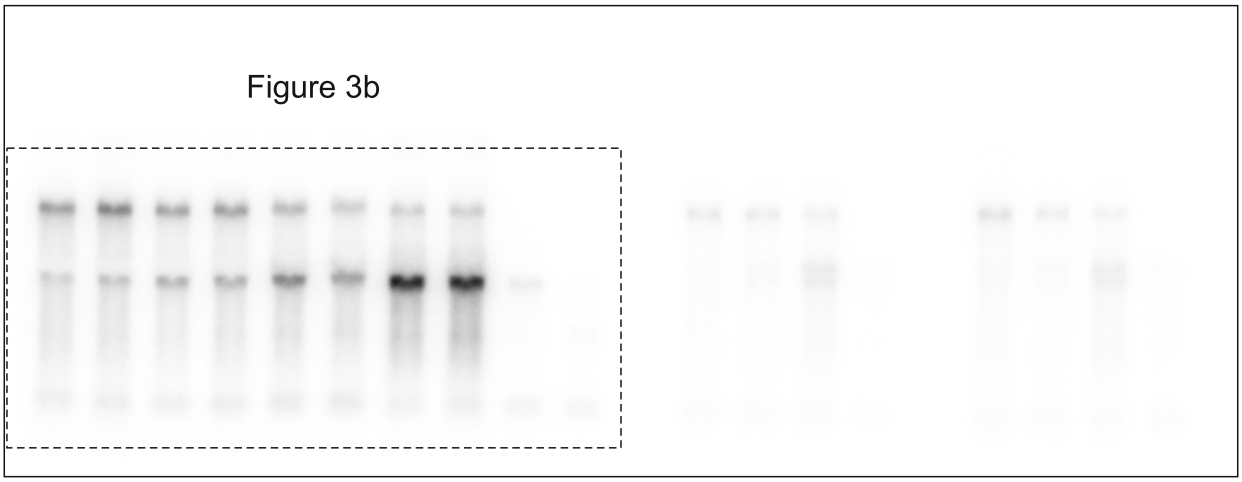

Supplementary Figure 1, continued

Uncropped blots of Figures 1-5 as indicated.

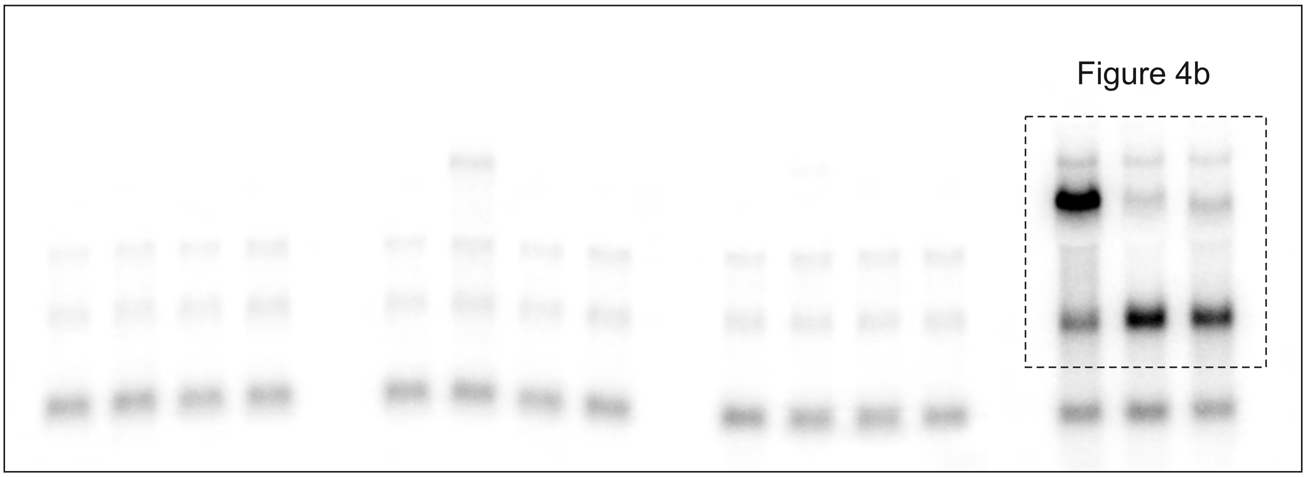

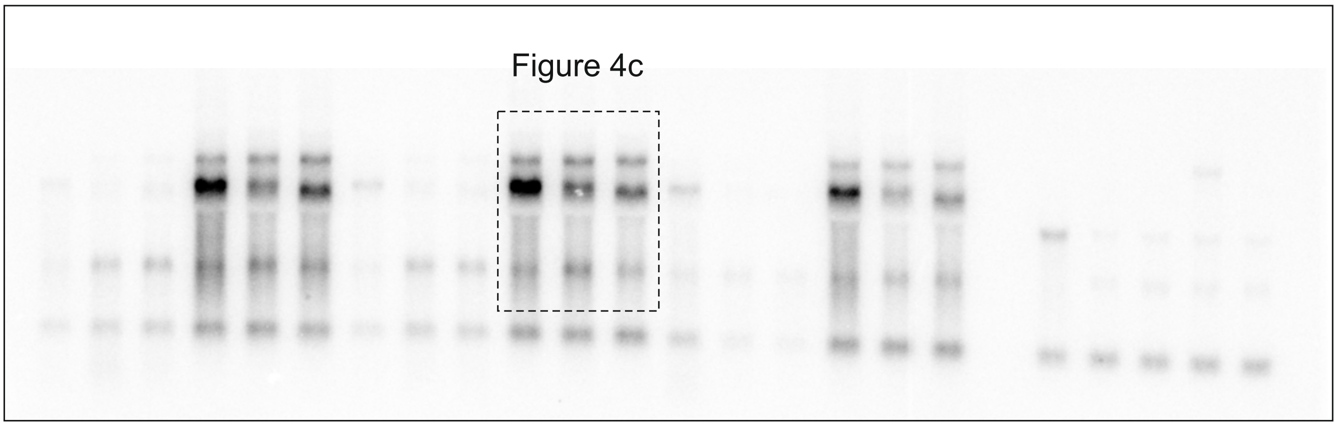

Supplementary Figure 1, continued

Uncropped blots of Figures 1-5 as indicated.

Figure 5a

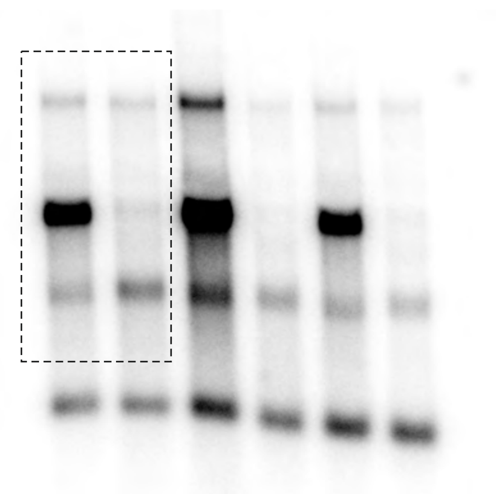

Figure 5b

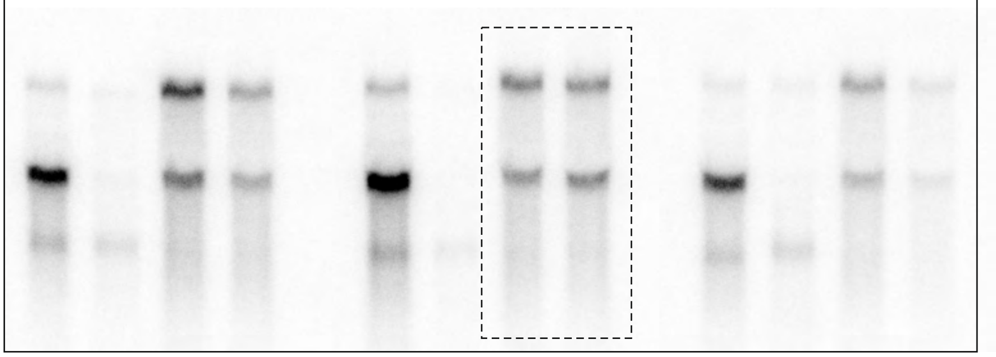

Figure 5c

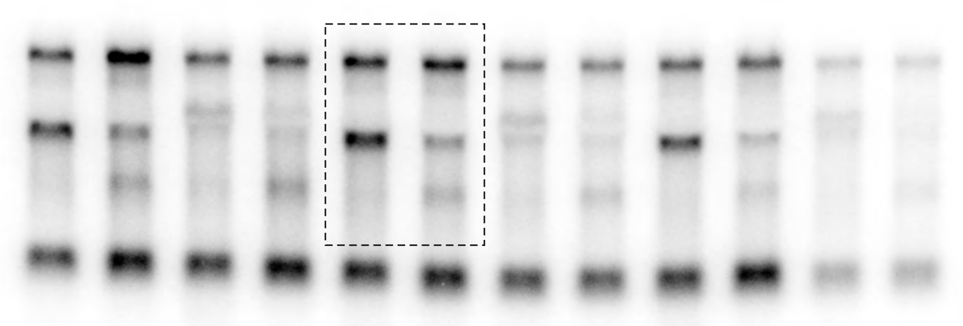

Figure 5d

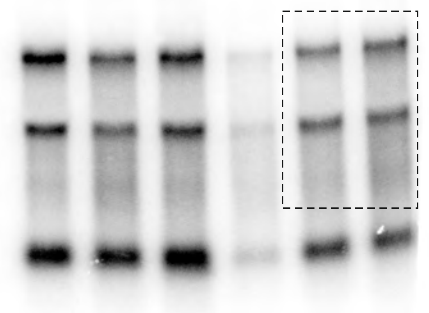

Supplementary Figure 1, continued

Uncropped blots of Figures 1-5 as indicated.
